# Machine learning classifier is associated with mortality in interstitial lung disease: a retrospective validation study leveraging registry data

**DOI:** 10.1186/s12890-024-03021-w

**Published:** 2024-05-23

**Authors:** Kavitha C. Selvan, Joshua Reicher, Michael Muelly, Angad Kalra, Ayodeji Adegunsoye

**Affiliations:** 1 Section of Pulmonary and Critical Care Medicine, Department of Internal Medicine, University of Chicago Medicine, 5841 S Maryland Avenue, Chicago, IL 60637 USA; 2 Department of Radiology, Stanford University, Stanford, CA USA; 3 IMVARIA Inc, 2390 Domingo Ave. #1496, Berkley, CA 94705 USA

**Keywords:** Interstitial lung disease, Machine learning, Mortality

## Abstract

**Background:**

Mortality prediction in interstitial lung disease (ILD) poses a significant challenge to clinicians due to heterogeneity across disease subtypes. Currently, forced vital capacity (FVC) and Gender, Age, and Physiology (GAP) score are the two most utilized metrics in prognostication. Recently, a machine learning classifier system, Fibresolve, designed to identify a variety of computed tomography (CT) patterns associated with idiopathic pulmonary fibrosis (IPF), was demonstrated to have a significant association with mortality across multiple subtypes of ILD. The purpose of this follow-up study was to retrospectively validate these findings in a large, external cohort of patients with ILD.

**Methods:**

In this multi-center validation study, Fibresolve was applied to chest CT scans of patients with confirmed ILD that had available follow-up data. Fibresolve scores categorized by tertile were analyzed using Cox regression analysis adjusted for tobacco use and modified GAP (mGAP) score.

**Results:**

Of 643 patients included, 446 (69.3%) died over a median follow-up time of 144 [1-821] weeks. The median [range] mGAP score was 5 [3–7]. In multivariable analysis, Fibresolve score categorized by tertile was significantly associated with mortality (Tertile 2 HR 1.47, 95% CI 0.82–2.37, *p* = 0.11; Tertile 3 HR 3.12, 95% CI 1.98–4.90, *p* < 0.001). Subgroup analyses revealed significant associations amongst those with non-IPF ILDs (Tertile 2 HR 1.95, 95% CI 1.28–2.97, Tertile 3 HR 4.66, 95% CI 2.94–7.38) and severe disease, defined by a FVC ≤ 75% (Tertile 2 HR 2.29, 95% CI 1.43–3.67, Tertile 3 HR 4.80, 95% CI 2.93–7.86).

**Conclusions:**

Fibresolve is independently associated with mortality in ILD, particularly amongst patients with non-IPF ILDs and in those with severe disease.

**Supplementary Information:**

The online version contains supplementary material available at 10.1186/s12890-024-03021-w.

## Introduction

Mortality in patients with interstitial lung disease (ILD) is highly variable, and providing accurate prognostic guidance to patients remains a significant challenge. The forced vital capacity (FVC) and Gender, Age, and Physiology (GAP) score are two metrics with proven prognostic value in idiopathic pulmonary fibrosis (IPF) and other ILDs [1–5]. Additionally, certain chest computed tomography (CT) features such as radiologic honeycombing, the hallmark feature of IPF, have been found to predict outcomes across a diverse spectrum of ILDs [6, 7]. However, the predictive value of other radiologic features that cannot be identified by visual assessment, such as texture or intensity distribution, remains uncertain.

In recent years, there has been a growing interest in the use of computer-based CT analysis to identify imaging features associated with outcomes in ILD. High-attenuation areas (i.e. the percentage of lung voxels between − 600 and − 250 Hounsfield units) and data-driven textural analysis have demonstrated associations with mortality and pulmonary function, respectively [8–10]. Fibresolve (IMVARIA, Berkeley, CA) is a proprietary software system that was designed as a non-invasive adjunct to diagnosing IPF through identification of a variety of associated CT features, including a usual interstitial pneumonia (UIP) pattern, in cases of uncertainty. Its utility in diagnosing UIP has been validated using internal and external multinational datasets, and more recently, has been expanded to prognostication in diverse subtypes of ILD within a small internal dataset (manuscript under review) [11].

The purpose of this study was to retrospectively validate the use of Fibresolve as a prognostic tool within a large external dataset of patients with diverse subtypes of ILD.

## Methods

### Study setting

This study leveraged data from a subset of patients included in a large international registry of approximately 3,000 patients with ILD collected between 2005 and 2020. The registry includes data from both public and nonpublic sources [12]. Public data sources included data provided by the Lung Tissue Research Consortium (LTRC) supported by the National Heart, Lung, and Blood Institute (NHLBI), and the Open Source Imaging Consortium (OSIC). Key metrics including patient demographic information, medical history, ILD diagnosis, and imaging were collected from the electronic medical record. Information on patient outcomes, including mortality and hospitalization, was also obtained. We consulted extensively with Argus Institutional Review Board (IRB) who determined that our study did not need ethical approval. An IRB official waiver of ethical approval was granted from Argus IRB. The data protocols are in accordance with the ethical standards of our institution and with the 1964 Helsinki declaration and its later amendments or comparable ethical standards. Informed consent was obtained from all participants.

### Previous development of the Fibresolve model

Fibresolve is a convolutional neural network (CNN) trained to assess patterns in 3-D volumetric CT scans [9]. Fibresolve was originally designed to analyze CT images of patients with suspected IPF in the pre-invasive setting and predict the final diagnosis. Importantly, Fibresolve assesses for a constellation of imaging features, including those that that define the UIP pattern associated with IPF; this broad assessment facilitates diagnosis in challenging IPF cases, in which probable UIP or indeterminate patterns may exist. This algorithm was trained using a multi-center database of over 2,000 confirmed cases of ILD and tuned using a US-based multi-site cohort of 295 cases. No data on medical treatment were included in model design or assessment.

### Study population and design

Using a subset of the larger databank previously unseen by the model and with no overlap in clinical sites (i.e. a hold-out test set), we performed a retrospective analysis of all patients with a physician-documented diagnosis of ILD, including: IPF, connective tissue disease-associated ILD (CTD-ILD), hypersensitivity pneumonitis (HP), unclassifiable ILD (UILD), and other ILDs. Patients receiving treatment with antifibrotic medications were not included, as the goal of this study was to assess baseline predictive performance of the model, uninfluenced by treatment (Figure E-1). All images were analyzed using Fibresolve. Model inputs included CT images in Digital Imaging and Communications in Medicine (DICOM) format, without inclusion of demographic or clinical data. The CT scans were made available from the database and varied across all major CT manufacturers, numerous individual scanner models, a range of reconstruction kernels, and up to 2.5 mm maximum slice thickness. Fibresolve’s raw assessment system (i.e. prior to final locking and calibration) was originally designed to evaluate combinations of chest CT patterns correlated with a pathologic and multi-disciplinary diagnosis of IPF through generation of a raw output metric (i.e. score) of 0 (meaning “very unlikely association with IPF”) or 1 (meaning “very likely association with IPF”) [11]. In a previously performed post-hoc analysis of 228 patients over a median follow-up period of 2.8 years (manuscript in review), Fibresolve’s added capacity to correlate with mortality, independent of ILD subtype, was evaluated through extraction of the raw score and found to be significantly associated with mortality in multivariable analysis (HR 7.14; 95% CI 1.31–38.85; *p* = 0.02).

### Follow-up and study outcomes

Patients entered the study on day of registry enrollment and were followed until the end of the study period, or loss to follow-up. Patients were censored if alive at the end of the study period, or at time of loss to follow-up. The outcome of interest, mortality, was quantified and compared across Fibresolve tertiles.

### Statistical analysis

Baseline patient characteristics were summarized using descriptive statistics and presented as medians with ranges or counts with proportions, as appropriate. The primary study outcome of mortality was assessed using Cox proportional hazard regression analyses, adjusted for potential confounders with an established relationship with ILD mortality chosen a-priori. These included age, sex, FVC, tobacco use, and a modified Gender, Age, and Physiology (mGAP) score (range − 2 to 8, with higher scores representing worse prognosis). The diffusion capacity of carbon monoxide (DLCO) percent, a component of the GAP score, was not available in the registry and an assumed value of + 3 (meaning “unable to perform”) was input to create the mGAP score. The mGAP score was classified as both a continuous variable and categorically by stage (stage I-III). Fibresolve score was classified as a categorical variable based on tertiles (1–3) in order to account for variance in continuous score scales (which can be deceptive in hazard ratio analysis) and to provide the most easily analyzed comparison to GAP stage. Tertile thresholds were set based on the entire population of patients with fibrotic ILD to allow for increased prognostic specificity and broad application in patients with heterogenous subtypes of disease. In a separate subgroup analysis (Table E-1), tertile thresholds were re-defined using ILD subtype-specific thresholds to provide estimates of hazard based on individual disease subtypes. Table E-2 provides Fibresolve tertile thresholds for the full cohort and specific subgroups.

In terms of analytical interpretation, the hazard function resulting from the Cox proportional hazard analysis produces a range of values derived from the underlying risk score generated by the machine learning model. The tertile buckets represent clustering of the ranked hazard positions for individual patients, which in turn reflect relative differences in the underlying risk score from the model, relative to the original patient distribution. Incorporating the input from a new patient outside of the analyzed dataset is conceptually similar to placing the patient within the same dataset and estimating their relative position with respect to risk profile. In this sense, results for new patients, as is true for any retrospective Cox proportional hazard analysis applied to patients outside the initially stratified dataset, should be interpreted with some degree of caution, as there is no guarantee that the new patient specifically falls within the same distribution as the original. For this reason, including wide ranges of patient outcomes in the original distribution increases likelihood of broad generalizability for use with future patient risk assessments. We have also included both univariate and multivariable approaches to the Cox proportional hazard analyses in order to: (1) provide both options in terms of attempting to compare performance to existing tools reported in the literature, and (2) make clear the differences in results of these separate analyses.

Additionally, survival curves were plotted using the Kaplan-Meier survival estimator, and the log-rank test was used for group comparisons. All analyses were conducted using IBM SPSS Statistics (Version 28.0).

## Results

### Patient characteristics

This study included 643 patients with a diagnosis of ILD. The median time from initial assessment (including CT scan) to final follow-up was 144 [IQR 1-821] weeks (Table 1). The median age was 66 years, and 63% were male. The median mGAP score was 5 [3–7]. The most common ILD subtype was IPF (*n* = 317, 49.3%), followed by CTD-ILD (*n* = 175, 27.22%) and UILD (*n* = 80, 12.44%). See Table E-3 for baseline characteristics stratified by Fibresolve tertile.

**Table 1:** Baseline demographic and clinical characteristics

Characteristic	Survived (*n* = 446)	Died (*n* = 197)	Total (*n* = 643)	*p*-value
Age – median (range)	64 (56–71)	70 (62–75)	66 (57–72)	< 0.001
Male – n (%)	280 (62.7)	123 (62.4)	403 (62.6)	0.50
Follow-up time (weeks) – median (IQR)	133 (1-821)	162 (1-684)	144 (1-821)	0.49
FVC (%) – median (range)	79.5 (21–153)	72.0 (26–132)	77.2 (21–153)	< 0.001
Tobacco use – n (%)	270 (61.5)	109 (57.1)	379 (60.2)	0.61
ILD Subtype – n (%)				
IPF CTD-ILD Unclassifiable HP Other^*^	251 (56.3) 111 (24.9) 38 (8.5) 14 (3.1) 32 (7.2)	74 (37.6) 63 (32.0) 42 (21.3) 11 (5.5) 7 (3.6)	325 (50.5) 174 (27.2) 80 (12.4) 25 (3.9) 39 (6.1)	
mGAP score – median (range)	5 (3–7)	5 (3–7)	5 (3–7)	< 0.001
mGAP stage – n (%)				
I II III	77 (17.3) 301 (67.5) 68 (15.2)	10 (5.1) 115 (58.4) 62 (31.5)	87 (13.5) 416 (64.7) 130 (20.2)	0.002 0.08 < 0.001

Key: FVC = forced vital capacity; ILD = interstitial lung disease; IPF = idiopathic pulmonary fibrosis, CTD-ILD = connective tissue disease-associated ILD; HP = hypersensitivity pneumonitis, GAP = Gender, Age, and Physiology

^*^“Other” ILD’s included idiopathic nonspecific interstitial pneumonia (iNSIP), bronchiolitis obliterans, desquamative interstitial pneumonia (DIP), exposure/iatrogenic ILD, and pleuroparenchymal fibroelastosis (PPFE)

p-value < 0.05 is statistically significant

### Univariate analysis

Fibresolve tertile 3 was significantly associated with mortality compared to Fibresolve tertile 1 (HR 2.28, 95% CI 1.55–3.34, *p* < 0.001). See Table 2. Additionally, age (HR 1.07, 95% CI 1.04–1.09, *p* < 0.001) and FVC (HR 0.98, 95% CI 0.96–0.99, *p* < 0.001) were significantly associated with mortality.

**Table 2:** Univariate analysis of predictors for mortality in ILD

Predictor	Hazard Ratio	95% Confidence Interval	*p*-value
Age	1.07	1.04–1.09	< 0.001
Sex	1.45	0.89–1.47	0.74
FVC (%)	0.98	0.96–0.99	< 0.001
Tobacco use	1.05	0.75–1.47	0.79
mGAP score	1.15	0.89–1.47	0.29
mGAP stage			
I II III	1.0 (Reference) 1.71 1.85	- 0.85–3.43 0.79–4.33	- 0.13 0.16
Fibresolve tertile			
I II III	1.0 (Reference) 1.41 2.28	- 0.98–2.04 1.55–3.34	- 0.07 < 0.001

Key: FVC = forced vital capacity; mGAP = modified Gender, Age, and Physiology

*p*-value < 0.05 is statistically significant

### Subgroup analysis of Fibresolve tertiles

Additional univariate analyses were completed in order to assess differences in Fibresolve performance by ILD subtype and baseline disease severity, defined by FVC % (Table 3). Fibresolve was significantly associated with mortality amongst patients with non-IPF diagnoses (Tertile 2 HR 1.95, 95% CI 1.28–2.97, Tertile 3 HR 4.66, 95% CI 2.94–7.38) and severe baseline disease (Tertile 2 HR 2.29, 95% CI 1.43–3.67, Tertile 3 HR 4.80, 95% CI 2.93–7.86).

**Table 3:** Univariate analysis of Fibresolve tertile and mortality by ILD subtype and baseline FVC

Subgroup	Hazard Ratio	95% Confidence Interval
IPF		
I II III	1.0 (Reference) 1.25 1.82	- 0.50–3.13 0.76–4.31
Non-IPF		
I II III	1.0 (Reference) 1.95 4.66	- 1.28–2.97 2.94–7.38
*CTD-ILD*		
I II III	1.0 (Reference) 1.95 4.66	- 1.28–2.97 2.94–7.38
*UILD*		
I II III	1.0 (Reference) 1.51 2.55	- 0.69–3.33 1.19–5.45
*CHP*		
I II III	1.0 (Reference) 0.79 5.82	- 0.09–7.23 1.12–30.19
Patients with FVC ≤ 75%		
I II III	1.0 (Reference) 2.29 4.80	- 1.43–3.67 2.93–7.86
Patients with FVC > 75%		
I II III	1.0 (Reference) 1.55 3.00	- 0.88–2.75 1.79–5.02

Key: CTD-ILD = connective tissue disease-associated interstitial lung disease; FVC = forced vital capacity; IPF = idiopathic pulmonary fibrosis; CHP = chronic hypersensitivity pneumonitis; UILD = unclassifiable interstitial lung disease

Note: Fibresolve tertile thresholds were set based on the full cohort of patients with ILD. Separate univariate analyses were run for each of the subgroups presented above

### Survival cut-off at five years

**Fig. 1:**
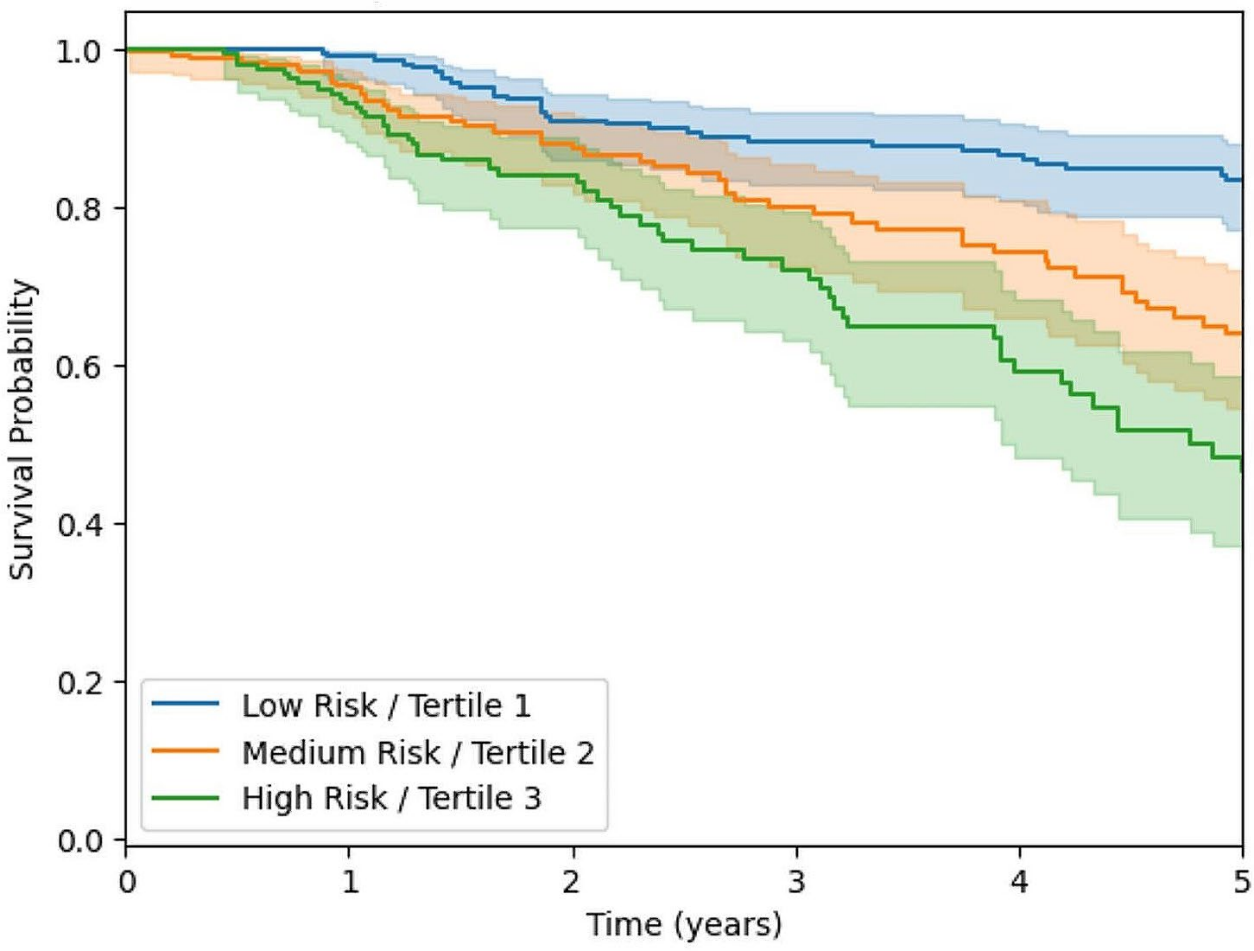
Kaplan -Meier 5-year survival by Fibresolve tertile

Further analysis with Kaplan-Meier survival curves found a significant difference in 5-year survival between Fibresolve tertiles. See Fig. 1. Log rank tests between groups found that all groups were significantly different (Tertile 1 versus Tertile 2, *p* < 0.0001; Tertile 1 versus Tertile 3, *p* < 0.0001; Tertile 2 versus Tertile 3, *p* = 0.005; or with Bonferroni corrections, Tertile 1 versus Tertile 2, *p* = 0.0002; Tertile 1 versus Tertile 3, *p* < 0.0001; Tertile 2 versus Tertile 3,  *p* = 0.01). Additional analysis of both mGAP and Fibresolve raw scores were completed to determine the area under the receiver operating characteristic curve (AUROC) in predicting mortality at 5 years, with an AUROC for mGAP as 0.50 (95% CI 0.43–0.56) and Fibresolve as 0.59 (95% CI 0.51–0.67).

### Multivariable analysis of Fibresolve tertiles

In a multivariable analysis adjusted for age, sex, FVC, and tobacco use, Fibresolve tertile 3 was associated with an increased risk of death (HR 2.45, 95% CI 1.54–3.89, *p* < 0.001) compared to Fibresolve tertile 1 (reference). See Table 4. In subsequent models adjusted for tobacco and mGAP score or mGAP stage, Fibresolve tertile 3 remained significantly associated with mortality (HR 3.12, 95% CI 1.98–4.90, *p* < 0.001 and HR 3.47, 95% CI 2.22–5.43, *p* < 0.001, respectively). When adjusted for mGAP stage, Fibresolve tertile 2 was also significantly associated with mortality when compared to Fibresolve tertile 1 (HR 1.62, 95% CI 1.01–2.59, *p* = 0.05).

**Table 4:** Multivariable analysis of the association between Fibresolve tertile and mortality in ILD

**Model A**	**Hazard Ratio**	**95% Confidence Interval**	***p-value***
**Fibresolve tertile**			
I	1.0 (Reference)	-	-
II	1.2	0.75–1.95	0.45
III	2.45	1.53–3.89	< 0.001
**Age**	1.08	1.06–1.10	< 0.001
**Sex**	0.99	0.69–1.42	0.96
**FVC (%)**	0.97	0.96–0.98	< 0.001
**Tobacco use**	1.03	0.73–1.44	0.87
**Model B**	**Hazard Ratio**	**95% Confidence Interval**	**p-value**
**Fibresolve tertile**			
I	1.0 (Reference)	-	-
II	1.47	0.82–2.37	0.11
III	3.12	1.98–4.90	< 0.001
**mGAP score**	1.96	1.67–2.29	< 0.001
**Tobacco use**	1.02	0.77–1.36	0.90
**Model C**	**Hazard Ratio**	**95% Confidence Interval**	**p-value**
**Fibresolve tertile**			
I	1.0 (Reference)	-	-
II	1.62	1.01–2.59	0.05
III	3.47	2.22–5.43	< 0.001
**mGAP stage**			
I	1.0 (Reference)	-	-
II	3.81	1.99–7.23	< 0.001
III	9.24	4.65–18.37	< 0.001
**Tobacco use**	0.97	0.75–1.33	0.98

Key: FVC = forced vital capacity; mGAP = modified Gender, Age, and Physiology

No input data were missing in the above multivariable analysis

*p*-value < 0.05 is statistically significant

Descriptions: Model A analyzes Fibresolve tertiles in a multivariate analysis with age, sex, FVC, and tobacco use. Model B analyzes Fibresolve tertiles in a multivariate analysis with mGAP score and tobacco use. Model C analyzes Fibresolve tertiles in a multivariate analysis with mGAP stage and tobacco use

## Discussion

In this international, retrospective validation study, we demonstrate that Fibresolve is significantly associated with mortality in ILD, particularly amongst patients with non-IPF ILDs in whom prognostication is most challenging. Fibresolve consistently maintained its mortality association when analyzed by disease severity, as determined by baseline FVC, underscoring the potential inherent value of this classifier algorithm amongst patients with severe fibrotic lung disease.

Accurate prediction of survival within ILD poses a significant challenge to clinicians due to the variable course seen across different disease subtypes. Various demographic, clinical, radiologic, and histologic factors have been identified as significant predictors of disease progression and survival. Among those, FVC and GAP score are the most routinely utilized. Numerous studies have demonstrated a strong association between mortality and both baseline FVC and FVC decline, particularly amongst patients with IPF [1–3]. The GAP score was subsequently developed in 2012 as a baseline mortality prediction model in IPF, accounting for patient gender, age, and lung physiology as assessed by FVC and DLCO [4]. Since that time, it has been applied to other subtypes of ILD with mixed data on prognostic accuracy, suggesting that its value remains highest amongst patients with IPF [5, 13, 14]. In our study, we found that both baseline FVC and Fibresolve tertile were significantly associated with mortality in both univariate and multivariable analyses. In this cohort of patients, mGAP score was not correlated with mortality in univariate analysis, though it was correlated with mortality in multivariable analyses. The cause of this finding is uncertain but may possibly be explained by the large number of non-IPF ILDs in this dataset, given the GAP score’s historical validation in IPF populations. When included in multivariable models, the mGAP score increased the strength of association between Fibresolve score and mortality, suggesting that the inclusion of these routinely available demographic and physiologic predictors may add value to Fibresolve.

Though FVC and GAP score provide powerful mortality prediction in IPF, there is still a need for more accurate tools for prognostication in non-IPF ILDs. In a subgroup analysis of ILD subtype and disease severity, Fibresolve’s association with mortality was strongest amongst patients with non-IPF diagnoses and in those with severe disease (defined as FVC ≤ 75%). Though the effect estimate did not reach significance, Fibresolve demonstrated a trend toward increased mortality in patients with IPF, as well. As expected, when Fibresolve tertile thresholds were re-defined using ILD subtype-specific thresholds, as opposed to a population-based thresholds, associations in IPF became significant. This can likely be explained by higher Fibresolve scores in patients with IPF. Though subtype-specific tertile thresholds bore similar results to population-based tertile thresholds, we believe the inherent value of Fibresolve is its utility in a real-world population of patients with fibrotic ILDs, in which subtype may not be certain.

In addition to identifying important clinical predictors of mortality, there has been a growing interest in the use of computer-based CT analysis to identify imaging features associated with outcomes in ILD. Several features derived by the computer algorithm, CALIPER, have been found to have prognostic value in IPF. These include CALIPER-measured pulmonary vascular volume (HR 1.23), volume of reticular abnormalities (HR 1.91), and volume/percent change of interstitial changes (HR 1.70 and 1.52, respectively) [15–17]. Notably, the features derived from these CALIPER studies are associated with lower hazard ratios than Fibresolve and again, were evaluated only in patients with IPF, limiting the generalizability of these findings. Additionally, these features were pre-specified rather than organically identified by the algorithm, which limits the evaluation of features that may not be identified by visual assessment. In contrast, deep learning algorithms such as Fibresolve are able to identify patterns in multidimensional datasets without human input, which allows for the identification of additional “hidden” or novel features that have not previously been evaluated [18].

When compared to other deep learning algorithms developed within ILD, such as Systematic Objective Fibrotic Imaging Analysis (SOFIA) [19, 20], Fibresolve allows for prognostication in ILD, independent of radiologic pattern. While both SOFIA and Fibresolve were initially designed to predict the probability of IPF, SOFIA was trained to recognize features consistent with a UIP pattern while Fibresolve recognizes a constellation of features beyond those associated with a UIP pattern [8, 19, 20]. This design feature may explain its demonstrated association with mortality in non-IPF ILDs, including CTD-ILD. Additionally, the hazard ratios for mortality associated with SOFIA were lower than what we report with use of Fibresolve (HR 1.75, 95% CI 1.37–2.25, *p* < 0.001 vs. Fibresolve tertile 3 HR 3.12, 95% CI 1.98–4.90, *p* < 0.001 in a multivariable model adjusted for tobacco use and GAP score, respectively). It is important to note, however, that a direct comparison between Fibresolve and CALIPER or SOFIA were not made in this study.

The impact of this study is not without limitations. First, it was performed retrospectively using registry data that did not include several covariates with known associations with ILD mortality, such as DLCO, radiologic/pathologic pattern of disease, and presence of pulmonary hypertension and/or lung cancer. Second, the DLCO percent was not available, so an input value of + 3 for DLCO was used for all patients in generating the mGAP score. Notably, only relative values are relevant in hazard ratio calculations, so there is no statistical impact of this approach to the results of the analyses themselves. We leveraged a real-world dataset in which DLCO values were not consistently obtained or routinely reported. The lack of inclusion of DLCO is subject to consideration, as it is a known predictor of mortality; however, DLCO is also limited by: (1) poor specificity, (2) limited reliability and reproducibility compared to FVC, (3) limited added prognostic value due to general correlation with FVC in ILD patients, and (4) difficulty to obtain the measurement, compared to FVC [21]. DLCO is known, however, to assist with assessments for secondary disorders including pulmonary hypertension, and these disorders were not specifically assessed in this study. Further assessments of relative utility of DLCO as an added variable could be of value. Third, the diagnostic certainty of ILD subtype was limited, as diagnoses were determined by review of physician-completed registry forms that did not include supportive documentation or recording of consistent methodology (e.g. gold-standard multidisciplinary diagnosis) [22]. As a result, we elected to exclude subtype from the primary analyses due to concerns about inconsistency and variability; however, we included subtype in secondary analyses. In the secondary analyses, though there was a trend toward significance, Fibresolve was not associated with mortality in the IPF subgroup when tertile thresholds were defined by the entire population. However, when subtype-specific tertile thresholds were used, Fibresolve was significantly associated with mortality in IPF, suggesting that higher thresholds are required for patients with IPF. Fourth, patients included in the registry were not on antifibrotic medications, which have a demonstrated association with FVC decline and outcomes in ILD [23–25]. Fifth, as occurs with the use of any deep learning algorithm, the exact imaging parameters scored by Fibresolve are unknown. Finally, this study utilized cross-sectional imaging, and longitudinal changes in Fibresolve score were not linked to outcomes given the study design. We demonstrate that combining Fibresolve output with non-imaging features, such as mGAP score, strengthens the association with mortality, and in the future, adding additional non-imaging variables such as pathology may prove to be a superior approach [26].

## Conclusion

 We demonstrate that Fibresolve is significantly associated with mortality in ILD, particularly amongst patients with non-IPF subtypes of disease. Routine application of this technology may ultimately improve disease prognostication in patients with non-IPF ILDs.

### Electronic supplementary material

Below is the link to the electronic supplementary material.

Supplementary Material 1

## Data Availability

The data supporting the findings of this study are available from the corresponding author upon reasonable request.

## Abbreviations

CT: Computed tomography
CTD-ILD: Connective tissue disease-associated interstitial lung disease
DICOM: Digital imaging and communications in medicine
DLCO: Diffusion capacity of carbon monoxide
DTA: Data-driven textural analysis
FVC: Forced vital capacity
GAP: Gender-Age-Physiology
HAA: High attenuation areas
HP: Hypersensitivity pneumonitis
ILD: Interstitial lung disease
IPF: Idiopathic pulmonary fibrosis
MESA: Multi-Ethnic Study of Atherosclerosis
mGAP: Modified Gender-Age-Physiology
OSIC: Open Source Imaging Consortium
PVV: Pulmonary vascular volume
SOFIA: Systematic Objective Fibrotic Imaging Analysis
UILD: Unclassifiable interstitial lung disease
UIP: Usual interstitial pneumonia
US: United States

## References

[1] Molina MM, Hart E, Lesher B, Ribera A, Langley J, Patel H (2023). Association between FVC and mortality in idiopathic pulmonary fibrosis: a systematic literature review. Eur Respir J.

[2] Chen X, Guo J, Yu D, Jie B, Zhou Y (2021). Predictors of mortality in progressive fibrosing interstitial lung disease. Front Pharmacol.

[3] Gimenez A, Storrer K, Kuranishi L, Soares MR, Ferreira RG, Pereira CAC (2018). Change in FVC and survival in chronic hypersensitivity pneumonitis. Thorax.

[4] Ley B, Ryerson CJ, Vittinghoff E, Rya JH, Tomassetti S, Lee JS (2012). A multidimensional index and staging system for idiopathic pulmonary fibrosis. Ann Intern Med.

[5] Reyerson CJ, Vittinghoff E, Ley B, Lee JS, Mooney JJ, Jones KD (2014). Predicting survival across chronic interstitial lung disease: the ILD-GAP model. Chest.

[6] Adegunsoye A, Oldham JM, Bellam SK, Montner S, Churpek MM, Noth I (2019). Computed tomography honeycombing identifies a progressive fibrotic phenotype with increased mortality across diverse interstitial lung diseases. Annals Am Thorac Soc.

[7] Brown KK, Inoue Y, Flaherty KR, Martinez FJ, Cottin V, Bonella F (2022). Predictors of mortality in subjects with progressive fibrosing interstitial lung diseases. Respirology.

[8] Podolanczuk AJ, Oelsner EC, Barr RG, Hoffman EA, Armstrong HF, Austin JHM (2016). High attenuation areas on chest CT in community-dwelling adults: the MESA study. Eur Respir J.

[9] Podolanczuk AJ, Oelsner EC, Barr RG, Bernstein EJ, Hoffman EA, Easthausen IJ (2017). High attenuation areas on chest computed tomography and clinical respiratory outcomes in community-dwelling adults. Am J Respir Crit Care Med.

[10] Humphries SM, Yagihashi K, Huckleberry J, Rho B-H, Schroeder JD, Strand M (2017). Idiopathic pulmonary fibrosis: data-driven textural analysis of extent of fibrosis at baseline at 15-month follow-up. Radiology.

[11] Ahmad Y, Mooney JJ, Allen I, Seaman J, Kalra A, Muelly M et al. A machine learning system to predict diagnosis of idiopathic pulmonary fibrosis non-invasively in challenging cases. Available at SSRN: https://ssrn.com/abstract=4353892 or 10.2139/ssrn.4353892 10.3390/diagnostics14080830 PMC11049625 38667475

[12] Maddali M, Kalra A, Muelly M, Reicher J (2023). Development and validation of a CT-based deep learning algorithm to augment non-invasive diagnosis of idiopathic pulmonary fibrosis. Respir Med.

[13] Low S (2015). Using the ILD-GAP model to predict mortality in chronic interstitial lung disease. Eur Respir J.

[14] Brusca RM, Pinal-Fernandez I, Psoter K, Paik JJ, Albayda J, Mecoli C (2019). The ILD-GAP risk prediction model performs poorly in myositis-associated interstitial lung disease. Resp Med.

[15] Jacob J, Bartholmai BJ, Rajagopalan S, Kokosi M, Nair A, Karwoski R et al. Mortality prediction in idiopathic pulmonary fibrosis: evaluation of computer-based CT analysis with conventional severity measures. Eur Respir J. 2017;49(1). 10.1183/13993003.01011-2016 27811068

[16] Jacob J, Bartholmai BJ, Rajagopalan S, van Moorsel CHM, van Es HW, van Beek FT (2018). Predicting outcomes in idiopathic pulmonary fibrosis using automated computed tomographic analysis. Am J Respir Crit Care Med.

[17] Maldonado F, Moua T, Rajagopalan S, Karwoski RA, Raghunath S, Decker PA (2014). Automated quantification of radiologic patterns predicts survival in idiopathic pulmonary fibrosis. Eur Respir J.

[18] Walsh SL, Humphries SM, Wells AU, Brown KK (2020). Imaging research in fibrotic lung disease; applying deep learning to unsolved problems. Lancet Respir Med.

[19] Walsh SLF, Calandriello L, Silva M, Sverzellati N (2018). Deep learning for classifying fibrotic lung disease on high-resolution computed tomography: a case-cohort study. Lancet Respir Med.

[20] Walsh SLF, Mackintosh JA, Calandriello L, Silva M, Sverzellati N, Larici AR (2022). Deep learning-based outcome prediction in progressive fibrotic lung disease using high-resolution computed tomography. Am J Respir Crit Care Med.

[21] Taha N, D’Amato D, Hosein K, Ranalli T, Sergiacomi G, Zompatori M, Mura M (2020). Longitudinal functional changes with clinically significant radiographic progression in idiopathic pulmonary fibrosis: are we following the right parameters?. Respir Res.

[22] Flaherty KR, King TE, Raghu G, Lynch JP, Colby TV, Travis WD (2004). Idiopathic interstitial pneumonia: what is the effect of a multidisciplinary approach to diagnosis?. Am J Respir Crit Care Med.

[23] Richeldi L, de Bois RM, Raghu G, Azuma A, Brown KK, Costabel U (2014). Efficacy and safety of nintedanib in idiopathic pulmonary fibrosis. N Eng J Med.

[24] Flaherty KR, Wells AU, Cottin V, Devaraj A, Walsh SLF, Inoue Y (2019). Nintedanib in progressive fibrosing interstitial lung disease. N Eng J Med.

[25] King TE, Bradford WZ, Castro-Bernardini S, Fagan EA, Glaspole I, Glassberg MK (2014). A phase 3 trial of pirfenidone in patients with idiopathic pulmonary fibrosis. N Eng J Med.

[26] Uegami W, Bychkov A, Ozasa M, Uehara K, Kataoka K, Johkoh T (2022). Mixture of human expertise and deep learning – developing an explainable model for predicting pathological diagnosis and survival in patients with interstitial lung disease. Mod Path.

