# Supplementary material for: Machine learning classifier is associated with mortality in interstitial lung disease: a retrospective validation study leveraging registry data
Source: BMC Pulm Med. 2024 May 23;24:254. doi: 10.1186/s12890-024-03021-w (PMC11112769; doi:10.1186/s12890-024-03021-w)
Supplement: Supplementary file 1 — Supplementary Material 1 [file 12890_2024_3021_MOESM1_ESM.docx]

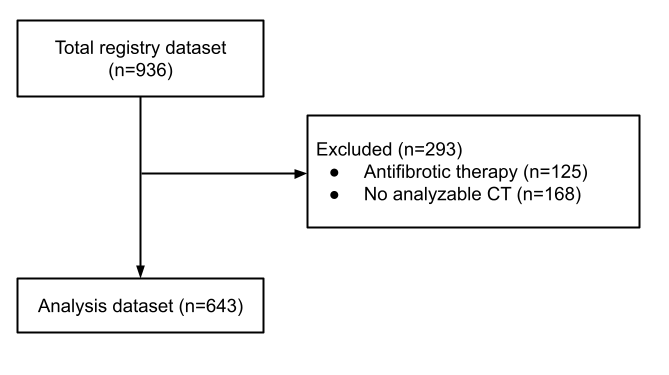
**Supplementary Material:**

**Figure E-1:** Study consort diagram

**Table E-1**: Univariate Analysis of Subtype-Specific Fibresolve Tertile and Mortality

| Subgroup | Hazard Ratio | 95% Confidence Interval |
| --- | --- | --- |
| IPF  I  II  III | 1.0 (Reference)  2.24  2.00 | -  1.20-4.18  1.09-3.70 |
| Non-IPF  I  II  III | 1.0 (Reference)  1.86  3.99 | -  1.13-3.06  2.48-6.41 |
| *CTD-ILD*  I  II  III | 1.0 (Reference)  2.12  4.83 | -  1.03-4.34  2.45-9.52 |
| *UILD*  I  II  III | 1.0 (Reference)  1.29  2.60 | -  0.56-2.97  1.19-5.69 |
| *CHP*  I  II  III | 1.0 (Reference)  2.80  3.90 | -  0.51-15.55  0.65-23.63 |
| Patients with FVC ≤75%  I  II  III | 1.0 (Reference)  1.81  4.64 | -  1.09-2.99  2.85-7.55 |
| Patients with FVC >75%  I  II  III | 1.0 (Reference)  1.97  3.29 | -  1.14-3.41  1.95-5.54 |

Key: CTD-ILD = connective tissue disease-associated interstitial lung disease; FVC = forced vital capacity; IPF = idiopathic pulmonary fibrosis; UILD = unclassifiable interstitial lung disease.

Note: Fibresolve tertile thresholds were set based on the subgroup-specific population of interest (i.e. based on ILD subtype or disease severity)

**Table E-2:** Fibresolve tertile cutoff values

| Group | Tertile 1/2 | Tertile 2/3 |
| --- | --- | --- |
| Full Cohort | 51 | 62 |
| IPF | 59 | 67 |
| Non-IPF | 51 | 62 |
| CTD-ILD | 40 | 54 |
| UILD | 47 | 58 |
| CHP | 36 | 51 |
| Patients with FVC ≤75% | 50 | 61 |
| Patients with FVC >75% | 53 | 64 |

Note: Fibresolve score is on a 0-100 scale.

**Table E-3:** Demographic and Clinical Characteristics by Fibresolve Tertile

| Characteristic | Tertile 1 (n=215) | Tertile 2 (n=214) | Tertile 3 (n=214) | p-value |
| --- | --- | --- | --- | --- |
| Age – median (range) | 59 (48-68.5) | 67 (59-72) | 64 (70-75) | <0.0001 |
| Male – n (%) | 103 (47.9) | 144 (62.3) | 156 (72.9) | <0.0001 |
| Follow-up time (weeks) – median (IQR) | 296 (162-442.5) | 100 (66-255) | 66 (89-201) | <0.0001 |
| FVC (%) – median (range) | 74.0 (21-132) | 75.8 (28-153) | 75.0 (22-146) | 0.01 |
| Tobacco use – n (%) | 47.9 (103) | 64.5 (138) | 64.5 (138) | 0.0001 |
| ILD Subtype – n (%)  IPF  CTD-ILD  Unclassifiable  HP  Other^*^ | 29 (13.4)  105 (48.8)  36 (16.7)  17 (7.9)  28 (13.0) | 129 (60.2)  48 (22.4)  27 (12.6)  4 (1.9)  6 (2.8) | 167 (78.0)  21 (9.8)  17 (7.9)  4 (1.9)  5 (2.3) | <0.0001  <0.0001  0.02  0.002  <0.0001 |
| mGAP score – median (range) | 4 (3-7) | 5 (3-7) | 5 (3-7) | 0.0002 |
| mGAP stage – n (%)  I  II  III | 43 (20%)  145 (67%)  27 (13%) | 25 (12%)  136 (63%)  53 (25%) | 19 (9%)  145 (68%)  50 (23%) | 0.002  0.48  0.003 |
